# Canadian public perceptions and experiences with information during the COVID-19 pandemic: strategies to optimize future risk communications

**DOI:** 10.1186/s12889-023-15659-y

**Published:** 2023-04-28

**Authors:** Suvabna Theivendrampillai, Jeanette Cooper, Taehoon Lee, Michelle Wai Ki Lau, Christine Marquez, Sharon E. Straus, Christine Fahim

**Affiliations:** Knowledge Translation Program, Unity Health Toronto, Toronto, Canada

**Keywords:** Knowledge dissemination, Misinformation, Health communication, Qualitative research, COVID-19

## Abstract

**Background:**

The COVID-19 pandemic accelerated the spread of misinformation worldwide. The purpose of this study was to explore perceptions of misinformation and preferred sources of obtaining COVID-19 information from those living in Canada. In particular, we sought to explore the perceptions of East Asian individuals in Canada, who experienced stigma related to COVID-19 messaging.

**Methods:**

We conducted a qualitative thematic analysis study. Interviews were offered in English, Mandarin and Cantonese. Interviewers probed for domains related to knowledge about COVID-19, preferred sources of information, perceived barriers and facilitators of misinformation, and preferences for communication during a health emergency. Interviews were recorded, translated, transcribed verbatim and analyzed using a framework approach. Transcripts were independently double-coded until > 60% agreement was reached. This study received research ethics approval.

**Results:**

Fifty-five interviews were conducted. The majority of participants were women (67%); median age was 52 years. 55% of participants were of East-Asian descent. Participants obtained information about COVID-19 from diverse English and non-English sources including news media, government agencies or representatives, social media, and personal networks. Challenges to seeking and understanding information included: encountering misinformation, making sense of evolving or conflicting public health guidance, and limited information on topics of interest. 65% of participants reported encountering COVID-19  misinformation. East Asian participants called on government officials to champion messaging to reduce stigmatizing and racist rhetoric and highlighted the importance of having accessible, non-English language information sources. Participants provided recommendations for future public health communications guidance during health emergencies, including preferences for message content, information messengers, dissemination platforms and format of messages. Almost all participants preferred receiving information from the Canadian government and found it helpful to utilize various mediums and platforms such as social media and news media for future risk communication, urging for consistency across all platforms.

**Conclusions:**

We provide insights on Canadian experiences navigating COVID-19 information, where more than half perceived encountering misinformation on platforms when seeking COVID-19 information . We provide recommendations to inform public health communications during future health emergencies.

**Supplementary Information:**

The online version contains supplementary material available at 10.1186/s12889-023-15659-y.

## Background

Public health information and guidance rapidly evolved during the COVID-19 pandemic. The novelty of the virus and lack of evidence enabled opportunities for misinterpretation of data and for the sharing of inaccurate information [1].

The spread of false information (i.e., misinformation), during the COVID-19 pandemic continues to be a global concern [2]. Misinformation and conspiracy theories arise during states of uncertainty and fear because of attempts to make sense of these situations [3, 4]. Misinformation can overshadow accurate information, challenging individuals to distinguish fact from fiction. In contrast, disinformation is the process of deliberately spreading false information. Both misinformation and disinformation have been prevalent across communication channels including social media [5–8], news media [7–10] and government officials [10, 11] during the COVID-19 pandemic.

Misinformation and disinformation have direct consequences on the public’s perception of risk and adherence to preventive health behaviours [9, 12, 13]. During the H1N1 outbreak in 2009, global news media did not communicate consistently about the risk and severity of infection [14]. These news media often de-emphasized the health risks H1N1 presented by using vague wording and lowered the public’s sense of urgency to take preventative actions [14, 15]. Canadian news media displayed similar patterns during COVID-19, with misinformation and disinformation driving reduced engagement in preventative behaviours including masking, physical distancing and vaccine uptake [9, 16].

In Canada and elsewhere, misinformation and disinformation also led to the stigmatization of certain populations, including individuals of East-Asian descent [17]. In partnership with racial advocacy organizations (the Chinese Canadian National Council Toronto Chapter (CCNCTO) and the Chinese Canadian National Council for Social Justice (CCNC-SJ)), we aimed to describe the perceptions and experiences of East-Asian and non-East Asian residents of Canada during the COVID-19 pandemic. We aimed to understand where participants obtained COVID-19 information, challenges experienced while navigating COVID-19 information, perceptions of misinformation, and where possible, its correlation with stigma. We also aimed to identify suggested strategies to mitigate misinformation and stigmatization during future public health emergencies.

## Methods

We report our study methods and findings using the Consolidated criteria for Reporting Qualitative Research (COREQ) checklist [18–23].

### Research Team and Reflexivity

Interviews were conducted by research staff experienced in qualitative methodology (TL (BSc at time of interviewing), CM (BSc), JC (MSc), MKL (BA). Three interviewers were women of White and racialized ethnicities and one interviewer was a man of East Asian ethnicity; MKL conducted interviews in Mandarin or Cantonese while the remaining interviewers conducted interviews in English. Women scientists of non-East Asian ethnicity (CF (PhD, MSc), SES (MD, MSc)) oversaw interview guide development and reviewed content to help determine saturation. Members of the interview team included those who arrived to Canada as immigrants and those who faced experiences of stigma and discrimination (in particular, racialized stigma towards East Asian people); these experiences may have placed a bias or influence on the data collection. In addition, the research team holds expertise in public health, epidemiology and health systems research, which may have influenced our views of COVID-19 related public health policy. Potential biases were mitigated by (1) having predefined questions and prompts to limit scope creep and support a neutral interviewing style and (2) use of a collaborative approach that facilitated our team to reflect on potential biases. Research personnel did not hold any relationships with the study participants. Participants were told about the objectives of the research and that the work was being conducted in partnership with community advocacy organizations; the participants did not have any additional knowledge of the interviewers. We used an integrated knowledge translation (iKT) approach to conduct this study [19–22], meaning that our study objectives and interpretation were following discussions with Chinese-Canadian advocacy organizations. Our partner organizations (CCNCTO, CCNC-SJ) noted experiences and concerns of racial and ethnic bias and discrimination towards Chinese and East-Asian individuals in Canada during the COVID-19 pandemic and highlighted the importance of messaging and communications during the pandemic to reduce stigma. It was through this lens that we shaped our research questions and developed our sampling approach. The results presented in this study are a subset of the interview data collected. Data describing participants’ experiences and perceptions of stigma during the COVID-19 pandemic are reported elsewhere.

### Study Design

#### Methodological orientation and theory

We conducted a qualitative, narrative study using semi-structured interviews with members of the public [18]. Data were analyzed using content analysis.

#### Participant selection

Participants were recruited via convenience and snowball sampling through advertisements on social media (Twitter, Kijiji) and newsletters through Unity Health Toronto and its networks. The CCNCTO and CCNC-SJ distributed recruitment flyers to their community members, who then shared it to group discussions on WeChat. We targeted recruitment to ensure equal representation of those who did and did not identify as East Asian. Recruitment materials were offered in English and simplified and traditional Chinese characters. Interviews were offered in English, French, Mandarin and Cantonese.

We aimed to recruit a minimum of 15 East Asian and 15 non-East Asian participants; however, we continued interviewing until data saturation in each subgroup was reached. We iteratively assessed participant demographics to ensure we reflected diversity by age, targeting individuals between 18 and 75 years old. An honorarium of a $25.00 CAD Visa gift card was provided to participants at the end of the interview.

#### Setting

Prior to their interview, participants were invited to complete a demographic questionnaire provided in English, French, traditional or simplified Chinese characters through Qualtrics survey platform [24]. Demographic data such as gender, age, occupation, region of residence, and education were collected. Survey completion was voluntary and responses were used to ensure that a diverse sample was recruited. Interviews were conducted between May 2020 – December 2020, representing the first and second waves of COVID-19 in Canada [25]. All interviews were conducted by telephone and participants verbally consented to participate and be recorded. Interviewers conducted the calls in a private space.

#### Data collection

A semi-structured interview guide was developed. Interview questions covered five domains including knowledge about COVID-19, preferred sources for COVID-19 information, perceived drivers and facilitators of misinformation, perceptions on COVID-19 guidance and policies, and recommendations for public health communications in future health emergencies (Additional File 1). There were no repeat interviews conducted. Following participant consent, interviews were audio recorded, de-identified and assigned participant identification numbers. Field notes were also taken by research staff during interviews and used to supplement and clarify concepts during analysis. Transcripts were not returned to participants.

### Analysis and findings

The Framework analysis approach was utilized to prepare and analyze the data [26]. English interviews were transcribed using NVivo transcription, an automated transcription service. Three research staff (TL, CM, and ST) reviewed, revised and verified the transcripts to ensure accuracy. Mandarin and Cantonese interviews were transcribed verbatim by the CCNCTO and translated to English. These transcripts were reviewed and verified by a research staff member to ensure accuracy and correct English interpretation (MKL).

A preliminary coding tree was developed by the research team using the research questions. Two team members (TL and CM) independently piloted the coding tree against three transcripts. A consensus meeting was then conducted whereby both members reviewed their findings and resolved any discrepancies. Additional categories were subsequently added to develop a final coding tree. Definitions for each of the codes were developed [26]. The remaining transcripts were then independently double coded in rounds using NVivo 11 software by research staff (TL, CM, JC, and ST) [27]. Each round of coding consisted of five transcripts and a consensus meeting was held between pairs of coders after each round. During the consensus meeting, any coded sections that did not have at least a 60% kappa agreement, which represents moderate level of agreement, were discussed [28]. Kappa was generated via the NVivo “coding comparison query” function. Inconsistencies between the two coders were reviewed, discussed and resolved prior to moving onto the next round, which facilitated improved Kappa with each iteration of coding. As coding progressed, iterations to the framework were made to build on, refine and improve the categories. The coding tree was used for interviews conducted in English, Mandarin and Cantonese, with respective modifications made to capture themes from each interview set.

Coded data in each category were charted into a framework matrix outlining thematic categories and references to illustrative quotations. Research staff (TL, CM, AP, JC, ST) charted the coded data. The matrix was used to explore theoretical concepts, map connections between categories and phenomena, and develop descriptive statistics. Participants did not provide feedback on the findings.

### Research ethics approval

This study received research ethics approval prior to initiation by the Research Ethics Board at Unity Health Toronto (study number 20–092).

## Results

A total of 55 interviews were conducted between May – December 2020; 29 interviews were conducted in English, 17 in Mandarin and nine in Cantonese. Four participants answered the initial recruitment ad but chose not to participate; none of the participants dropped out of the study. The mean interview duration was 58.16 min. The average coding kappa value for the English language interviews was 0.910 and the average kappa value for the Chinese language interviews was 0.909.

### Demographics

The median sample age was 52 years old (range: 20–76). Participants reported their gender as 67.3% women (n = 37), 30.9% men (n = 17) and 1.8% (n = 1) did not answer the question. Approximately half of the participants identified as East Asian (n = 30, 55%). Other participants identified as Caucasian (n = 17, 32%), South Asian (n = 4, 7%), LatinX (n = 1, 2%) and Black (n = 1, 2%). The majority of the participants had a university degree or higher, (n = 44, 80%) and were from Ontario, Canada (n = 43, 78%). See Table 1 for all demographic characteristics.

**Table 1:** Demographic Breakdown

Demographics	Number of individuals (n = 55)
**Gender**	
Men	17 (EN 18.0%, CN 13%)
Women	37 (EN 33%, CN 35%)
Unanswered	1
**Age**	
18–34	16 (EN 20%, CN 10%)
35–54	18 (EN 16%, CN 16%)
55–74	16 (EN 11%, CN 18%)
75 +	3 (EN 2%, CN 4%)
Unanswered	2
**Ethnicity**	
Black	1 (EN 2%)
LatinX	1 (EN 2%)
East Asian	30 (EN 7%, CN 47%)
South Asian	4 (EN 7%)
White	17 (EN 32%)
Prefer not to answer	1
Unanswered	1
**Education**	
12th grade or less	0
Graduated high school or equivalent	8 (EN 5%, CN 10%)
College	4 (EN 2%, CN 5%)
University Degree	25 (EN 22%, CN 24%)
Post-graduate Degree	19 (EN 20%, CN 15%)
Unanswered	2
**Province/Territory**	
British Columbia	1 (EN 2%)
Alberta	2 (EN 4%)
Saskatchewan	4 (EN 7%)
Manitoba	0
Ontario	43 (EN 33%, CN 45%)
Quebec	2 (EN 4%)
New Brunswick	0
Newfoundland & Labrador	0
Prince Edward Island	2 (EN 4%)
Nova Scotia	0
Yukon	0
Northwest Territories	0
Nunavut	0
**Region**	
Large urban population center, with a population of 100,000 or more	40 (EN 31%, CN 42%)
Medium population center, with a population between 30,000 and 99,999	8 (EN 10%, CN 5%)
Small population center, with a population between 1,000 and 29,99	3 (EN 5%)
Rural area, with a population less than 1,000	4 (EN 7%)
**Employment Status**	
Full Time	20 (EN 20%, CN 16%)
Part Time	6 (EN 4%, CN 7%)
Self employed	3 (EN 2%, CN 4%)
Not employed	9 (EN 8%, CN 10%)
Retired	13 (EN 15%, CN 10%)

### Information sources

Participants obtained COVID-19 information from various sources. News media, including print media, was the information source most commonly used by participants to obtain COVID-19 information (n = 38, 68%). Canadian and American news media were cited as information sources, including the Canadian Broadcasting Corporation (CBC), CityNews, Global News, Cable News Network (CNN), The Fox Broadcasting Company (FOX), OMNI Television, The Sun and Fairchild. In addition to English news media, East Asian participants relied on Chinese news sources originating from Mainland China and Hong Kong, such as Ming Pao or Sing Tao Daily for COVID-19 information. All participants aged 61 or older (n = 15, 27%) reported that news media was their preferred source for obtaining COVID-19 information.

Approximately one-third (n = 19, 35%) of participants relied on social media for COVID-19 information, specifically Facebook, Twitter and WhatsApp. In addition to these platforms, East Asian participants used WeChat to obtain COVID-19 information. Social media was frequently cited by all participants aged 61 years or younger (n = 19, 35%) as an information source.

Other popular information sources included government agencies and officials (n = 20, 36%), including Health Canada, the Public Health Agency of Canada, provincial health authorities, the Prime Minister of Canada, and the Chief Public Health Officer for public health updates. Some participants (n = 7, 13%) relied on their social networks for COVID-19 information including their friends, colleagues, family and community support workers. Four participants used websites to obtain COVID-19 information, particularly the World Health Organization (WHO), the Centers for Disease Control (CDC), and university websites. East Asian participants used Chinese-language websites originating from Mainland China (such as sina.com and youku.com), Hong Kong (such as Sing Tao Daily www.singtao.ca) and Canadian-based websites providing information in Chinese-language (such as 51.ca).

### Challenges seeking and understanding information

#### Perceptions of Misinformation

More than half of the participants (n = 36, 65%) reported encountering misinformation. Many shared that they did not expect to see the amount of misinformation that was circulating on media channels during the time of the pandemic, as demonstrated in the following quote,

“*I would have never thought there’d be as much misinformation out there as there is. It was quite surprising to me.*” *PT560*.

Participants reported seeing various unconfirmed theories related to the virus origins and misinformation related to strategies to prevent COVID-19 infections, as identified in the following quote,

*“Swallowing bleach, injecting bleach, just nonsense. We use bleach on our counter tops and that’s not nonsense, but it’s not injecting it. Hydroxychloroquine, I know he’s [Donald Trump] proposed and is on it, but I’ve listened to very reputable medical people saying there is no evidence for that and hydroxychloroquine does have potentially serious side effects.” PT63*

East-Asian participants also observed information related to the benefits of Chinese traditional medicines to protect against COVID-19. Participants shared their challenges differentiating “fact” from “fiction”. Strategies to interpret information and navigate misinformation included: only using sources they perceived as trusted information sources (most commonly cited as government or ‘official’ public health sources), cross-referencing information across sources to determine consistency, and using personal judgement, as seen in the following quotes,

*“I would believe articles that are based on evidence, that only talk about facts and are not emotional. I believe articles that source information from official sources, and talk about science and evidence. There are articles that are very emotional or written in a way to mislead and incite people. I can easily spot them and I won’t believe them. I would also see which media company the article is written by. Is it a small media company? Or does it have advertisements and is sponsored? I don’t trust those types of media. But if the media is official or scientific, then I will believe it.” PT692*

*“I see something that’s new about COVID, I’ll dig into it and check the source and check the facts and cross-reference before I will put it out there now. So I’m a lot more careful than I was in the beginning” – PT352*

#### Making sense of evolving or conflicting public health guidance

The novelty of COVID-19 resulted in rapidly changing scientific evidence and as such, participants noted seemingly contradictory information on COVID-19 guidelines and policies. Recommendations on protection and control measures changed between the first and second waves of the pandemic. Participants reported that this evolving guidance caused confusion and distrust.

The majority of participants shared frustrations with navigating evolving guidance, as seen in the following quotes,

*“The World Health Organization or CDC [Centers for Disease Control and Prevention] or both, they were saying, ‘don’t bother wearing masks, masks don’t do anything, masks aren’t effective, you’re wasting your time, don’t bother wearing them.’ And then within a few weeks, up until now, everyone is ‘masks, masks and masks’, which is a total contradiction.” PT108*

*“I do not totally believe the information published. Sometimes the information can be contradictory. For instance, [public health personnel] at first discouraged wearing masks, but later [public health personnel] encouraged wearing masks. There was no explanation as to why [public health personnel] changed [their] position. So, I don’t fully believe the media. My experiences in China, the US, and Canada have taught me that the media will always be biased, so I will do a lot of research myself. Some people may trust the media, but the media can be misleading.” PT295*

Other evolving evidence pertained to the virus pathology, epidemiology, illness severity, modes of transmission, and methods of treatment. The changing evidence fueled diverse, often polarized, conversations among the public, and led to the release of opposing theories and guidance, as demonstrated in the following quote,

*“And today, there are contradictory opinions on medicines to use for this virus. We see that the governments of different countries have different opinions on the effectiveness and side effects of different medicines. There are even different voices in the medical field, which really makes the public confused.” – PT144*

Furthermore, many participants perceived policies and directives from the Canadian government to be contradictory to guidance from other countries. Participants reported that country variances in COVID-19 communications and recommended preventive health measures were also confusing.

*“The Canadian Public Health Department hasn’t been clear on their policies and guidelines. From the beginning of the pandemic, to the middle, to the later stages where we are now, health measures and standards provided by leadership have constantly been changing. Their unclear communication creates hysteria among ordinary people.” PT534*

*“The most obviously confusing information is related to mask-wearing. I have family members in China, so I know that China made wearing a mask mandatory since the outbreak started. When the virus started to spread in Canada, at first mask wearing was not encouraged. I started to wear a mask rather early.” PT295*

#### Lack of information

In many cases, participants reported challenges with information seeking (see Table 2). Participants were challenged to identify credible information on how to recognize symptoms of COVID-19, infection prevention and control measures, local case numbers, treatments, vaccine development, and impact on systems (e.g., economy, healthcare system). Participants were not aware of relevant government public health policies and initiatives as they emerged, as identified in the following quote,

*“I always see people lining up outside testing centers for COVID test, and I always wonder if everyone can get tested or only people who work can get tested? Do they have to pay to get tested? I don’t know these policies, nor do people around me, so I hope there can be more information sharing on the policies.” PT694*

Moreover, participants felt that adequate information on new COVID-19 developments (e.g., vaccine development and availability) was not being communicated to the public in Canada,

*“Things like what is happening outside of Toronto, in Canada. When are things reopening? Where? Which ones? More about the vaccine. Like is it in development? What’s happening with it? I don’t know if they’re [News media such as CBC and CTV] being completely open with the public. They always say there’s a clinical trial but what’s exactly happening? It’s not very clear.” PT909*

The desire for additional a minority of participants (n = 5, 9%) felt they had too much information to navigate, as demonstrated in the following quote,

*“I think there is just such an overwhelming amount of information that it can be very overwhelming and hard to sift through.” PT009*

**Table 2:** Topics of interest participants had difficulty obtaining information on

Information Type	Examples
**Identifying Symptoms**	• Testing for COVID-19 • Navigating COVID-19 if infected • Contact tracing if exposed to COVID-19
**Pathology**	• Mutations to SARS-CoV-2
**Prevention & Control**	• Government policies • Implementation of policies • Legal consequences if public failed to follow policies
**Treatment Efforts**	• Development of treatment efforts and remedies to treat and cure COVID-19 • Development of the vaccine
**Impact on Global Systems**	• Impact of COVID-19 on the economy • Implications on specific populations (e.g. elderly) • Monitoring overall death counts and daily case numbers • Local impacts within hospitals and long-term care homes

### Recommendations for public health communications during a health emergency

#### Message content

Participants emphasized the importance of providing clear information pertaining to: epidemiology (e.g., transmission, COVID-19 case counts), preventive health measures (e.g., masking, physical distancing, infection prevention measures), illness characteristics, severity and health impact (e.g., on mental health), treatments (e.g., vaccines), and systems impact (e.g., employment, finances). Participants noted that public health messaging should evolve, as the pandemic evolves, and should address public concerns at that time, as noted in the following quote,

*“During different stages of the pandemic, there are different problems. There should not be merely numbers of infections and recovered patients. The government should communicate at a deeper level and talk about mental health, financial aid, infection prevention, and medication.” PT120*

Participants emphasized the importance of consistent, clear messaging and accountability from the government (including public health units), in particular. This was perceived as critical to fostering public trust, as demonstrated in the following quote,

*“Also, more collaboration with different provincial officers of health or chief medical officers around the messaging so that it is more clear and consistent across the board. Lack of consistency sends mix messaging to people. When it’s not consistent people don’t take it seriously.” PT55*

Participants also expressed a need for timely, rapid responses from the Canadian government to acknowledge the status of the pandemic, provide relevant guidance and implement directives to mitigate misinformation, uncertainty and stigmatization. Participants shared that the frequency of information should be commensurate with the severity of the pandemic (i.e., with more messages expected during times of increased risk/urgency, and a decrease of messaging as the risk/urgency decreases). Moreover, they requested positive messaging from the government to encourage the public and provide reassurance during situations of uncertainty. Participants aged 18–40 years perceived the government was not transparent, and recommended this be improved in future health communications. Some East Asian participants stressed the importance of government-led messaging to reduce stigmatization and discrimination, as noted in the following participant quotes. Additional recommendations on message content are provided in Table 3.

“*Yeah, I think it would be really good always to have someone like a medical officer of health or someone acknowledge that there’s people who are at high risk of being stigmatized. Any sort of this. I think that is very valuable, especially because I know a lot of people oftentimes value that the experts are coming from someone who you consider as an expert. Then it might make people realize that this is an issue that people will experience as a result of this. Yes, and acknowledge what those impacts are like, for example, if you think of the Chinese community, there’s been a lot of backlash against that community because there’s a lot of people who still believe that this is something that Chinese people here in Canada or anywhere are to blame. So just having someone say it is important that we recognize that this is not a reason we shouldn’t be treating people or stigmatizing them this way.*” PT 717

*“I think like a reaffirmation and like an increased emphasis on the fact that the Chinese people are not to blame for the disease and that Chinese Canadians don’t have an increased risk for the disease, and these things I think is important to highlight.” PT 009*.

**Table 3:** Key messages to address in future health communications

Key Messages	Examples
Epidemiology	• Case numbers (e.g. *number of people infected with COVID-19, number of people with confirmed COVID infection admitted to hospital, number of people recovering from COVID-19 infection, number of people with morbidity and mortality from COVID-19 infection*) • Location of outbreaks (e.g.*Sharing information on outbreaks in a specific residential area, outbreaks in a commercial center, outbreak in a retail location*) • Region specific data (*e.g. total number of new cases in a jurisdiction like x cases in Eastern Toronto*)
Symptoms, Transmission and Severity	• Symptoms (e.g. *what visible symptoms to be aware of if infected with COVID-19, like fever, flu, nausea*) • Modes of transmission (e.g.*how is COVID-19 transmitted, human to human, aerosol, airborne*) • Severity and impact (e.g.*seriousness of the pandemic, how many people have been impacted globally and locally*)
Prevention	• Mask wearing (e.g.*when should a mask be worn, what kind of masks should be used, how to safely use and dispose of a mask*) • Physical distancing (e.g. *when is social distancing appropriate, what is the appropriate distance*) • Public Health Practices (e.g.*what are different prevention methods, when should these methods be enforced and follow, which methods are more effective than others*)
Treatment and cure	• Current treatments (e.g.*treatment plans in place to treat people infected with COVID-19*) • Vaccine updates (e.g. *status on vaccine development, information on the different approaches that are being taken to research and create a vaccine globally*)
Impacts on other aspects	• Finances (e.g.*the impact of the pandemic on finances such as job security, closure of labor markets, difficulty finding new jobs, government aid like CERB*) • Global economy (e.g.*which countries are going in debt because of the pandemic and recovery, which countries cannot afford recovery for COVID-19, systems in place to support economic challenges globally*) • Mental Health (e.g.*available services for those experiencing depression and anxiety, coping strategies to deal with anxiety*) • Housing (e.g. *changes to housing, paying rent, subsidized housing*)
Policies and Directives	• New policies and guidance (e.g.*restrictions on public activities and centers, lockdown measures, mandated mask wearing and social distancing, government supports, enforcement policies and fines for public*)

#### Information messengers

Participants wanted to receive COVID-19 information from various sources, though almost all participants preferred receiving information from the Canadian government. Participants trusted information from all levels of government, including the federal, provincial, local governments and public health agencies and recommended these organizations disseminate consistent health messages.

Many participants believed that medical organizations (e.g., Canadian Medical Association), hospitals and academics should be responsible for disseminating information. Many participants cited trust in these institutions, particularly the researchers and medical experts (e.g., physicians) who worked there.

#### Dissemination platforms

Participants stated it would be helpful to use a range of platforms to tailor COVID-19 information to groups of diverse ages and ethnic backgrounds. Social media (e.g., Twitter, Instagram, Facebook, YouTube, WhatsApp and WeChat) was highlighted as a key platform for future dissemination. Participants over the age of 61 years cited the importance of using TV and radio to disseminate health information. East Asian participants cited the importance of Chinese-language platforms to disseminate health information and suggested there should be more platforms for the public to share questions/concerns about the pandemic. East Asian participants over the age of 61 recommended that physical flyers be placed in public settings, including malls, grocery stores and banks to disseminate health information. Despite the majority of participants citing the use of news sources as their preferred dissemination platform, only a minority believed news networks should be the key messengers during a public health emergency. Only two and three participants, respectively, believed health communications should be messaged by social media influencers or community centers.

#### Format of messages

Participants believed there should be variation in the format of messages disseminated. Participants thought the use of various mediums, including text, graphics, audio broadcast, and videos would be beneficial and emphasized the need to make information accessible, as seen in the following quote,

“*I found a visual graphic to be very helpful, and so I think anything that has color is like a flowchart is easy to sort of digest, makes it [the information] easier to engage with, it makes it [the information] more accessible.” PT009*

Participants aged 61 years or older appeared to prefer text communications whereas those aged 18–40 years preferred videos and visuals. Participants suggested that information be provided in multiple languages to increase accessibility.

## Discussion

Our study found that people living in Canada utilized various sources for COVID-19 related information. Consistent with other research, we found that the public had high levels of trust in government sources and medical officers, and recommended these individuals be the preferred messengers for public health information during health emergencies [8, 10]. We noted two major differences in perceptions and preferences between East-Asian and non-East-Asian participants in our sample.

First, East-Asian participants called on the Canadian government to champion messaging to reduce stigmatizing and racist rhetoric. Our study did not directly explore the link between exposure to misinformation and perceptions or experiences of stigmatization, yet we posit that the rise of stigmatizing information directly impacted the experiences of East Asian individuals living in Canada during the pandemic [29]. COVID-19 related information was often laden with stigmatizing messages, which were shared widely on mainstream and social media channels. This misinformation often included negative depictions of China and Chinese people, including prominent East-Asian public health officials who were the targets of racist attacks [30–34]. Participants also noted the need for government officials to recognize the ongoing stigmatization of racialized populations, such as Chinese Canadians, following the COVID-19 pandemic. Participants highlighted the importance of debunking misinformation, correcting stigmatizing language and maintaining transparency in communication [14–15].

Secondly, East Asian participants noted their reliance on accessible, non-English language data sources, particularly Chinese-language news media, websites and platforms that they used to obtain COVID-19 information. While our study focused on the experience of East Asian Canadians during the pandemic, the sentiments expressed are likely comparable for other ethnic, religious, and community groups [8, 10, 35–39]. Given the diversity of the Canadian population, additional research to identify such sources and develop plans to reach additional segments of the population is critical to ensuring accessible and equitable health messaging [10, 40].

With the exception of these two themes, the remaining perceptions and recommendations provided by participants were consistent across the East-Asian and Non-East-Asian participant groups. The majority of participants in our study obtained their information from news sources and print news media, while a minority cited use of American news media such as FOX, the New York Times or international sources such as the WHO. Similarly, Leigh and colleagues found that Canadians preferred Canadian sources over American or international sources [11]. Most participants in our study said their preferred information messengers were the Canadian government or scientific/medical institutions. Quantitative research conducted by our team showed similar trends [8, 10, 35–40]. In a survey of residents of Ontario, Canada, sources that were perceived to be highly trustworthy were less commonly used to obtain COVID-related information [41]. Similarly, while participants perceived social media to be a source of misinformation, 51% of our sample used it to obtain COVID-19 information. These results align with other research suggesting that individuals consume information from sources that they do not inherently trust [42]. Further, there is a need for additional research exploring the correlations between trust and use of information sources and digital literacy [43].

Participants reported encountering misinformation and often had trouble determining fact from fiction. This finding is consistent with other research exploring misinformation encountered by the global public during the pandemic, and highlights the prevalence of misinformation during COVID-19 [6, 8, 10, 44–47]. Another challenge was the lack of public trust caused by inconsistent or evolving messaging. This was particularly witnessed around messages pertaining to the benefits of masking. In Canada, the federal government initially advised against the use of masks among the public, suggesting they have limited benefits and supply was reserved for healthcare workers [48]. As the pandemic progressed however, guidance from the WHO advised public mask use in December 2020[49] and universal masking was significantly associated with decreased infection rates of COVID-19 [50]. The Canadian government then recommend the use of masks in May 2020, with many municipalities subsequently mandating mask-wearing. Participants viewed this guidance as conflicting and perceived this to be a source of misinformation spread [51–53].

Effective crisis and public health communication strategies are warranted to mitigate occurrences of misinformation [54]. Crisis communication should be reliable, messaged by trusted individuals who are able to debunk misinformation, and should clearly address uncertainty [6, 44, 55, 56]. Dissemination frameworks, such as the WHO’s Strategic Communications Framework, urge the importance of understanding socio-demographic and cultural characteristics of target populations, including communication preferences and acknowledging attitudes towards risk [54]. We use the WHO framework to interpret our study findings and provide recommendations for messaging in future public health emergencies. First, listening to public opinions and requests of information can provide verified data to the public’s questions are available and leaves less room for misinformation [54]. Our study showed that accurate and plain language information transmission, prevention strategies and treatment options was needed and seen as a gap in Canadian health communications. Participants also recommended that public health guidance be updated as the evidence evolves, in a transparent manner that accurately conveys levels of risk [55]. For instance, during the H1N1 pandemic, messages de-emphasizing H1N1 risk led to an imbalance of accurate information [14] and low perceived risk of infection among Australians [14].

Messages should address uncertainty to reduce the possibility of the public creating their own conclusions and thereby decreasing the potential for misinformation [55]. Additionally, public health messengers should take accountability for seemingly contradictory or conflicting information in order to maintain trust; this is particularly important among historically disadvantaged groups who may be less trusting of governments, media, health and academic institutions [7, 54]. Finally, the importance of ensuring information is accessible through various platforms, formats and languages was apparent in our data. Accessibility of data can be improved by providing messages in various languages or as both text or visuals to target varying literacy levels or engage younger populations [57]. Messages should also be consistent across news media, social media and print resources [15].

There are some limitations to this study. Participants were recruited through various channels, but mostly through internet and social media. This lacks a representative sample, as those who participated would have generally been active users of social media, which may present a bias in preferences. The respective reach and influence of these outlets need to be investigated in further research, including analyses by population sub groups (e.g. by race/ethnicity, gender or age). Our study population was highly educated with 80% having at least a bachelor’s degree or higher, which is significantly higher than the general Canadian population, and is a limitation given evidence showing correlations between levels of education and trust in political institutions [58, 59]. This sample was also skewed to individuals living in Ontario and the specific information sources cited may not be relevant to other regions in Canada; however, general experiences navigating COVID-19 information appear consistent across global literature [6, 8, 9, 11, 44, 56, 60]. This study was conducted as part of a larger program of research exploring the role of stigma and misinformation, particularly as it affected Chinese and East Asian Canadians during the pandemic. We therefore only purposefully recruited for ethnic representation among Chinese and East Asian groups, which does not represent the diversity of Canada or the information sources they leverage. Future research should further investigate similarities and differences among population subgroups, particularly to identify preferred news sources and messengers that can be leveraged to optimize public health communication during an emergency. Lastly, our study was conducted during the first and second waves of the pandemic and may not capture other important COVID-19 topics of interest (e.g., vaccine safety).

## Conclusion

The Canadian public used diverse sources to obtain COVID-19 information. Participants experienced significant challenges obtaining accurate guidance due to conflicting information, misinformation, evolving guidance and lack of information on topics of interest. East Asian participants highlighted the importance of public health communications to mitigate stigmatization and racist rhetoric and ensuring health messages are accessible to non-English language speakers. Participants provided suggestions on optimizing health messaging content and delivery to reduce misinformation and promote public trust during health emergencies.

## Electronic supplementary material

Below is the link to the electronic supplementary material.

Supplementary Material 1

## Data Availability

set and materials. The datasets generated and/or analyzed during the current study are not publicly available due privacy of participants but are available from the corresponding author on reasonable request.

## List of Abbreviations

CCNCTO: Chinese Canadian National Council Toronto Chapter
CCNC-SJ: Chinese Canadian National Council for Social Justice
COREQ: Consolidated criteria for Reporting Qualitative Research
CBC: Canadian Broadcasting Corporation
CNN: Cable News Network
FOX: The Fox Broadcasting Company
WHO: World Health Organization
CDC: Centers for Disease Control

## References

[1] Van Der Linden S, Roozenbeek J, Compton J. Inoculating Against Fake News About COVID-19. Front Psychol [Internet]. 2020 Oct 23 [cited 2022 Nov 11];11:2928. Available from: 10.3389/FPSYG.2020.566790/BIBTEX 10.3389/fpsyg.2020.566790 PMC7644779 33192844

[2] World Health Organization. Understanding the infodemic and misinformation in the fight against COVID-19 [Internet]. Available from: https://www.who.int/health-topics/infodemic/understanding-the-infodemic-and-misinformation-in-the-fight-against-covid-19#tab=tab_1

[3] Van Prooijen JW, Douglas KM. Conspiracy theories as part of history: The role of societal crisis situations. Mem Stud [Internet]. 2017 Jul 1 [cited 2022 Dec 19];10(3):323–33. Available from: 10.1177/1750698017701615 10.1177/1750698017701615 PMC5646574 29081831

[4] Bavel JJV, Baicker K, Boggio PS, Capraro V, Cichocka A, Cikara M et al. Using social and behavioural science to support COVID-19 pandemic response. Nat Hum Behav [Internet]. 2020 May 1 [cited 2022 Dec 19];4(5):460–71. Available from: 10.1038/s41562-020-0884-z 10.1038/s41562-020-0884-z 32355299

[5] Bridgman A, Merkley E, Loewen PJ, Owen T, Ruths D, Teichmann L et al. The causes and consequences of COVID-19 misperceptions: Understanding the role of news and social media. Harvard Kennedy School Misinformation Review [Internet]. 2020 Jun 18 [cited 2022 Dec 19]; Available from: 10.37016/mr-2020-028

[6] Fridman I, Lucas N, Henke D, Zigler CK. Association between public knowledge about COVID-19, trust in information sources, and adherence to social distancing: Cross-sectional survey. JMIR Public Health Surveill [Internet]. 2020 Jul 1 [cited 2022 Dec 19];6(3). Available from: 10.2196/22060 10.2196/22060 PMC7511226 32930670

[7] Al-Zaman MS. Prevalence and source analysis of COVID-19 misinformation of 138 countries. IFLA Journal [Internet]. 2021 [cited 2022 Dec 19];48(1):189–204. Available from: 10.1101/2021.05.08.21256879

[8] Lupton D, Lewis S. Learning about COVID-19: a qualitative interview study of Australians’ use of information sources. BMC Public Health [Internet]. 2021 Dec 1 [cited 2022 Dec 19];21(1). Available from: 10.1186/s12889-021-10743-7 10.1186/s12889-021-10743-7 PMC8024176 33823843

[9] Nagler RH, Vogel RI, Gollust SE, Rothman AJ, Fowler EF, Yzer MC. Public perceptions of conflicting information surrounding COVID-19: Results from a nationally representative survey of U.S. adults. PLoS One [Internet]. 2020 Oct 1 [cited 2022 Dec 19];15(10). Available from: 10.1371/journal.pone.0240776 10.1371/journal.pone.0240776 PMC7577476 33085719

[10] Ejaz W, Ittefaq M. Data for understanding trust in varied information sources, use of news media, and perception of misinformation regarding COVID-19 in Pakistan. Data Brief [Internet]. 2020 Oct 1 [cited 2022 Dec 19];32. Available from: 10.1016/j.dib.2020.106091 10.1016/j.dib.2020.106091 PMC7390753 32789157

[11] Leigh JP, Fiest K, Brundin-Mather R, Plotnikoff K, Soo A, Sypes EE et al. A national cross-sectional survey of public perceptions of the COVID-19 pandemic: Self-reported beliefs, knowledge, and behaviors. PLoS One [Internet]. 2020 Oct 1 [cited 2022 Dec 19];15(10). Available from: 10.1371/journal.pone.0241259 10.1371/journal.pone.0241259 PMC7584165 33095836

[12] Dryhurst S, Schneider CR, Kerr J, Freeman ALJ, Recchia G, van der Bles AM et al. Risk perceptions of COVID-19 around the world. J Risk Res [Internet]. 2020 [cited 2022 Dec 19];23(7–8):994–1006. Available from: 10.1080/13669877.2020.1758193

[13] Travis J, Harris S, Fadel T, Webb G. Identifying the determinants of COVID-19 preventative behaviors and vaccine intentions among South Carolina residents. PLoS One [Internet]. 2021 Aug 1 [cited 2022 Dec 19];16(8). Available from: 10.1371/journal.pone.0256178 10.1371/journal.pone.0256178 PMC8386860 34432817

[14] Sandell T, Sebar B, Harris N. Framing risk: Communication messages in the Australian and Swedish print media surrounding the 2009 H1N1 pandemic. Scand J Public Health [Internet]. 2013 [cited 2022 Dec 19];41(8):860–5. Available from: 10.1177/1403494813498158 10.1177/1403494813498158 23873631

[15] Chang C. Motivated Processing: How People Perceive News Covering Novel or Contradictory Health Research Findings. Sci Commun [Internet]. 2015 Oct 8 [cited 2022 Dec 19];37(5):602–34. Available from: 10.1177/1075547015597914

[16] Uscinski JE, Enders AM, Klofstad C, Seelig M, Funchion J, Everett C et al. Why do people believe COVID-19 conspiracy theories? Harvard Kennedy School Misinformation Review [Internet]. 2020 Apr 28 [cited 2022 Dec 19]; Available from: 10.37016/mr-2020-015

[17] Devakumar D, Shannon G, Bhopal SS, Abubakar I. Racism and discrimination in COVID-19 responses. The Lancet [Internet]. 2020 Apr 11 [cited 2022 Dec 19];395(10231):1194. Available from: 10.1016/S0140-6736(20)30792-3 10.1016/S0140-6736(20)30792-3 PMC7146645 32246915

[18] Creswell JW, Cheryl N. Poth. Qualitative Inquiry & Research Design. SAGE. SAGE; 2018. pp. 1–779.

[19] Gagliardi AR, Berta W, Kothari A, Boyko J, Urquhart R. Integrated knowledge translation (IKT) in health care: A scoping review. Implementation Science [Internet]. 2016 Mar 17 [cited 2023 Jan 5];11(1):1–12. Available from: https://implementationscience.biomedcentral.com/articles/10.1186/s13012-016-0399-1 10.1186/s13012-016-0399-1 PMC4797171 26988000

[20] Nguyen T, Graham ID, Mrklas KJ, Bowen S, Cargo M, Estabrooks CA et al. How does integrated knowledge translation (IKT) compare to other collaborative research approaches to generating and translating knowledge? Learning from experts in the field. Health Res Policy Syst [Internet]. 2020 Mar 30 [cited 2023 Jan 5];18(1):1–20. Available from: https://health-policy-systems.biomedcentral.com/articles/10.1186/s12961-020-0539-6 10.1186/s12961-020-0539-6 PMC7106699 32228692

[21] Jull J, Giles A, Graham ID. Community-based participatory research and integrated knowledge translation: Advancing the co-creation of knowledge. Implementation Science [Internet]. 2017 Dec 19 [cited 2023 Jan 5];12(1):1–9. Available from: https://implementationscience.biomedcentral.com/articles/10.1186/s13012-017-0696-3 10.1186/s13012-017-0696-3 PMC5735911 29258551

[22] Sharon S, Graham I, Harrison MB. Illustrating the knowledge to action cycle. Sharon Straus, Jacqueline Tetroe IDG, editor. Knowledge translation in health care: Moving from evidence to practice [Internet]. 2013 [cited 2023 Jan 5];249–62. Available from: https://www.wiley.com/en-sg/Knowledge+Translation+in+Health+Care%3A+Moving+from+Evidence+to+Practice%2 C+2nd+Edition-p-9781118413548

[23] COREQ. (Consolidated Criteria for Reporting Qualitative research) Checklist.

[24] Qualtrics. Qualtrics XM. The Leading Experience Management Software [Internet]. Available from: https://www.qualtrics.com/

[25] Detsky AS, Bogoch II. COVID-19 in Canada: Experience and Response to Waves 2 and 3. JAMA [Internet]. 2021 Sep 28 [cited 2022 Dec 19];326(12):1145–6. Available from: 10.1001/jama.2021.14797 10.1001/jama.2021.14797 34424275

[26] Gale NK, Heath G, Cameron E, Rashid S, Redwood S. Using the framework method for the analysis of qualitative data in multi-disciplinary health research. BMC Med Res Methodol [Internet]. 2013 Sep 18 [cited 2022 Nov 11];13(1):1–8. Available from: 10.1186/1471-2288-13-117/PEER-REVIEW 10.1186/1471-2288-13-117 PMC3848812 24047204

[27] About, NVivo. [Internet]. [cited 2023 Mar 20]. Available from: https://support.qsrinternational.com/s/article/TRC-About-NVivo-NVivo-11-for-Windows

[28] McHugh ML. Interrater reliability: the kappa statistic. Biochem Med (Zagreb) [Internet]. 2012 [cited 2023 Mar 20];22(3):276. Available from: /pmc/articles/PMC3900052/. PMC3900052 23092060

[29] Fahim C, Cooper J, Theivendrampillai S, Pham B, Straus SE. Exploring Canadian perceptions and experiences of stigma during the COVID-19 pandemic. Front Public Health [Internet]. 2023 Mar 7 [cited 2023 Mar 20];11:687. Available from: https://www.frontiersin.org/articles/10.3389/fpubh.2023.1068268/full 10.3389/fpubh.2023.1068268 PMC10027913 36960376

[30] Heng L. Chinese Canadians facing hate, racism for coronavirus outbreak — much like the SARS outbreak in 2003 | National Post. National Post [Internet]. 2020 Feb 1 [cited 2023 Mar 20]; Available from: https://nationalpost.com/news/chinese-canadians-facing-hate-racism-for-coronavirus-outbreak-much-like-the-sars-outbreak-in-2003

[31] Jeffords S. Ministers, mayor push back against coronavirus stigma in Toronto’s Chinatown neighbourhood - Toronto | Globalnews.ca. The Canadian Press [Internet]. 2020 Feb 11 [cited 2023 Mar 20]; Available from: https://globalnews.ca/news/6535457/toronto-mayor-provincial-federal-health-ministers-coronavirus/

[32] Labbé F, Pelletier C, Bettinger JA, Curran J, Graham JE, Greyson D et al. Stigma and blame related to COVID-19 pandemic: A case-study of editorial cartoons in Canada. Soc Sci Med. 2022 Mar 1;296:114803. 10.1016/j.socscimed.2022.114803 PMC8830930 35168055

[33] Rodriguez J, Coronavirus, CTV News. Urgent calls for feds to revitalize Canadian Chinatowns in light of anti-Asian racism |. 2021 Apr 12 [cited 2023 Mar 20]; Available from: https://www.ctvnews.ca/health/coronavirus/we-need-to-support-our-chinatowns-urgent-calls-for-feds-to-revitalize-hard-hit-areas-1.5384148

[34] Garneau K, Zossou C. Misinformation during the COVID-19 pandemic [Internet]. 2021 [cited 2023 Mar 20]. Available from: https://www150.statcan.gc.ca/n1/pub/45-28-0001/2021001/article/00003-eng.htm

[35] Umeta B, Mulugeta T, Mamo G, Alemu S, Berhanu N, Milkessa G et al. An analysis of COVID-19 information sources. J Pharm Policy Pract [Internet]. 2022 Dec 1 [cited 2022 Nov 11];15(1):1–9. Available from: 10.1186/S40545-022-00446-8/TABLES/7 10.1186/s40545-022-00446-8 PMC9383678 35978417

[36] Farooq A, Laato S, Islam AKMN, Isoaho J. Understanding the impact of information sources on COVID-19 related preventive measures in Finland. Technol Soc [Internet]. 2021 May 1 [cited 2022 Nov 11];65:101573. Available from: 10.1016/J.TECHSOC.2021.101573 10.1016/j.techsoc.2021.101573 PMC9754674 36540654

[37] Islam NS, Patel S, Wyatt LC, Sim SC, Mukherjee-Ratnam R, Chun K et al. Sources of Health Information among Select Asian American Immigrant Groups in New York City. Health Commun [Internet]. 2016 Feb 1 [cited 2022 Nov 11];31(2):207. Available from: 10.1080/10410236.2014.944332 10.1080/10410236.2014.944332 PMC4628554 26266574

[38] Bhatt N, Bhatt B, Gurung S, Dahal S, Jaishi AR, Neupane B et al. Perceptions and experiences of the public regarding the COVID-19 pandemic in Nepal: A qualitative study using phenomenological analysis. BMJ Open. 2020 Dec 12;10(12). 10.1136/bmjopen-2020-043312 PMC7735126 33310812

[39] Cui T, Yang G, Ji L, Zhu L, Zhen S, Shi N et al. Chinese residents’ perceptions of COVID-19 during the pandemic: Online cross-sectional survey study. J Med Internet Res [Internet]. 2020 Nov 1 [cited 2022 Dec 19];22(11). Available from: 10.2196/21672 10.2196/21672 PMC7690970 33152684

[40] Ali SH, Foreman J, Tozan Y, Capasso A, Jones AM, DiClemente RJ. Trends and Predictors of COVID-19 Information Sources and Their Relationship With Knowledge and Beliefs Related to the Pandemic: Nationwide Cross-Sectional Study. JMIR Public Health Surveillence [Internet]. 2020 Oct 8 [cited 2022 Dec 19];6(4):e21071. Available from: 10.2196/21071 10.2196/21071 PMC7546863 32936775

[41] Fahim C, Cooper J, Theivendrampillai S, Pham B, Straus S. Exploring Ontarians’ perceptions of and experiences with misinformation during the COVID-19 pandemic.J Med Internet Res. 2023 10.2196/38323 PMC10241226 37159394

[42] Tsfati Y, Cappella JN. Media Psychology Why Do People Watch News They Do Not Trust? The Need for Cognition as a Moderator in the Association Between News Media Skepticism and Exposure. Media Psychol [Internet]. 2010 Jun 24 [cited 2023 Mar 20];7:251–71. Available from: https://www.tandfonline.com/action/journalInformation?journalCode=hmep20

[43] Scherer LD, McPhetres J, Pennycook G, Kempe A, Allen LA, Knoepke CE et al. Who is susceptible to online health misinformation? A test of four psychosocial hypotheses. Health Psychol [Internet]. 2021 Mar 1 [cited 2023 Mar 20];40(4):274–84. Available from: https://pubmed.ncbi.nlm.nih.gov/33646806/10.1037/hea0000978 33646806

[44] Pickles K, Cvejic E, Nickel B, Copp T, Bonner C, Leask J et al. COVID-19 misinformation trends in Australia: Prospective longitudinal national survey. J Med Internet Res [Internet]. 2021 Jan 1 [cited 2022 Dec 19];23(1). Available from: 10.2196/23805 10.2196/23805 PMC7800906 33302250

[45] Ceron W, de-Lima-Santos MF, Quiles MG. Fake news agenda in the era of COVID-19: Identifying trends through fact-checking content. Online Soc Netw Media [Internet]. 2021 Jan 1 [cited 2022 Nov 11];21:100116. Available from: 10.1016/J.OSNEM.2020.100116

[46] Wang V, Liu SE, Fuller R, Cheng CI, Ragina N. Discerning Fact From Fiction: An Assessment of Coronavirus-19 Misinformation Among Patients in Rural Michigan. Cureus [Internet]. 2022 Jan 29 [cited 2022 Nov 11];14(1). Available from: 10.7759/CUREUS.21710 10.7759/cureus.21710 PMC8884155 35242476

[47] Sallam M, Dababseh D, Yaseen A, Al-Haidar A, Taim D, Eid H et al. COVID-19 misinformation: Mere harmless delusions or much more? A knowledge and attitude cross-sectional study among the general public residing in Jordan. PLoS One [Internet]. 2020 Dec 1 [cited 2022 Nov 11];15(12). Available from: 10.1371/JOURNAL.PONE.0243264 10.1371/journal.pone.0243264 PMC7714217 33270783

[48] Vogel L. Who should wear a face mask? Experts weigh in on Canada’s COVID-19 response. Can Med Assoc J [Internet]. 2020 Apr 20 [cited 2022 Dec 19];192(16):E440–1. Available from: 10.1503/cmaj.1095863 10.1503/cmaj.1095863 PMC7207188 32312831

[49] World Health Organization. Mask use in the context of COVID-19: interim guidance [Internet]. 2020 [cited 2023 Jan 17]. Available from: https://apps.who.int/iris/handle/10665/337199

[50] Wang X, Ferro EG, Zhou G, Hashimoto D, Bhatt DL. Association between Universal Masking in a Health Care System and SARS-CoV-2 Positivity among Health Care Workers. JAMA [Internet]. 2020 Aug 18 [cited 2022 Dec 19];324(7):703–4. Available from: 10.1001/jama.2020.12897 10.1001/jama.2020.12897 PMC7362190 32663246

[51] Hornik R, Kikut A, Jesch E, Woko C, Siegel L, Kim K. Association of COVID-19 Misinformation with Face Mask Wearing and Social Distancing in a Nationally Representative US Sample. Health Commun [Internet]. 2021 [cited 2022 Nov 11];36(1):6–14. Available from: 10.1080/10410236.2020.1847437 10.1080/10410236.2020.1847437 33225745

[52] Bok S, Martin DE, Acosta E, Lee M, Shum J, Bok S et al. Validation of the COVID-19 Transmission Misinformation Scale and Conditional Indirect Negative Effects on Wearing a Mask in Public. International Journal of Environmental Research and Public Health 2021, Vol 18, Page 11319 [Internet]. 2021 Oct 28 [cited 2022 Dec 19];18(21):11319. Available from: 10.3390/IJERPH182111319 10.3390/ijerph182111319 PMC8583109 34769835

[53] He L, He C, Reynolds TL, Bai Q, Huang Y, Li C et al. Why do people oppose mask wearing? A comprehensive analysis of U.S. tweets during the COVID-19 pandemic. Journal of the American Medical Informatics Association [Internet]. 2021 Jul 14 [cited 2022 Dec 19];28(7):1564–73. Available from: 10.1093/JAMIA/OCAB047 10.1093/jamia/ocab047 PMC7989302 33690794

[54] World Health Organization. WHO Strategic Communications Framework for Effective Communication [Internet]. Available from: https://www.who.int/docs/default-source/documents/communicating-for-health/communication-framework.pdf?sfvrsn=93aa6138_0

[55] Su Z, McDonnell D, Wen J, Kozak M, Abbas J, Šegalo S et al. Mental health consequences of COVID-19 media coverage: the need for effective crisis communication practices. Global Health [Internet]. 2021 Dec 1 [cited 2022 Dec 19];17(1):1–8. Available from: 10.1186/S12992-020-00654-4/FIGURES/1 10.1186/s12992-020-00654-4 PMC7784222 33402169

[56] Krause NM, Freiling I, Beets B, Brossard D. Fact-checking as risk communication: the multi-layered risk of misinformation in times of COVID-19. J Risk Res [Internet]. 2020 [cited 2022 Dec 19];23(7–8):1052–9. Available from: 10.1080/13669877.2020.1756385

[57] Person B, Sy F, Holton K, Govert B, Liang A, Garza B et al. Fear and Stigma: The Epidemic within the SARS Outbreak. Emerg Infect Dis [Internet]. 2004 [cited 2022 Dec 19];10(2):358. Available from: 10.3201/EID1002.030750 10.3201/eid1002.030750 PMC3322940 15030713

[58] Statistics Canada. Education Highlight Tables, 2016 Census [Internet]. 2016. Available from: https://www12.statcan.gc.ca/census-recensement/2016/dp-pd/hlt-fst/edu-sco/Tablecfm?Lang=E&T=11&Geo=00&View=2&Age=2

[59] Charron N, Rothstein B. Does education lead to higher generalized trust? The importance of quality of government. Int J Educ Dev [Internet]. 2016 Sep 1 [cited 2022 Nov 11];50:59–73. Available from: 10.1016/J.IJEDUDEV.2016.05.009

[60] Filkuková P, Ayton P, Rand K, Langguth J. What Should I Trust? Individual Differences in Attitudes to Conflicting Information and Misinformation on COVID-19. Front Psychol [Internet]. 2021 Jun 21 [cited 2022 Dec 19];12. Available from: 10.3389/fpsyg.2021.588478 10.3389/fpsyg.2021.588478 PMC8262492 34248728

