# Supplementary material for: Canadian public perceptions and experiences with information during the COVID-19 pandemic: strategies to optimize future risk communications
Source: BMC Public Health. 2023 Apr 28;23:796. doi: 10.1186/s12889-023-15659-y (PMC10141811; doi:10.1186/s12889-023-15659-y)
Supplement: Supplementary file 1 — Supplementary Material 1 [file 12889_2023_15659_MOESM1_ESM.docx]

**Additional File 1: Public Member Interview Guide**

**Legend**

File format is a word document, .doc. The title of the data is “Additional File 1: Public Member Interview Guide”. This file outlines the questions from the interview guide that probed for public knowledge of COVID-19, misinformation experiences and proposed recommendations for future messaging.

**Section 1: General Knowledge and Misinformation Experiences**

1. **What is your current knowledge about the novel coronavirus or COVID-19?**
   1. How did you first learn about COVID-19?
   2. Do you feel as if you have enough information on COVID-19?
   3. Is there any information about COVID-19 that is confusing? If so, can you describe?
2. **How have you been obtaining your information regarding COVID-19?**
   1. What type of information were you most interested in learning about COVID-19?
   2. What sources (e.g., online media, news channels, family and friends, etc.) have you been using to get your information?
   3. Are these the sources you typically use to obtain news/information?
   4. What challenges have you experienced in terms of seeking information about COVID?
   5. What has been helpful for you in terms of seeking information about COVID?
3. **Have you come across any misinformation regarding COVID-19? If so, can you please describe some examples of misinformation?**
   1. What would you say are typically the main sources of this misinformation?
   2. Origin of the virus (e.g., that it came from a research lab in China)
   3. Diagnostics, prevention, cure (e.g., consuming garlic is a cure)
   4. Transmission (e.g., that it is NOT transmitted by human-human contact)
   5. Severity of virus (e.g., that the flu is worst)
4. **Do you think there is someone to blame for misinformation?**
5. **What are your thoughts on how the public has reacted to the COVID-19 outbreak?**
   1. Do you agree with what you have observed?

**Section 2: Next Steps for Future Messaging**

1. **What key messages would you have liked have seen regarding the novel coronavirus or COVID-19? Did these key messages (that you would have liked to have seen) change over the course of the pandemic? If yes, can you provide examples?**
2. **Are there any key messages regarding stigma or fear that you would have liked to have seen regarding COVID-19?**
   1. How would the key messages need to be displayed for you to pay attention to them? (e.g., font, colour, flow of information, layout of information)
   2. How often would you want these key messages given to you?
3. **Who do you trust that could provide you with these key messages on COVID-19?**
4. **Who are specific individuals or organizations that should be disseminating key messages to the public?**
5. **How should they be disseminating these key messages on COVID-19?**
   1. Would you want these key messages delivered through text/audio clips/visuals/videos?
6. **How did you learn about this study?**
7. **Do you have any other feedback, comments, or concerns regarding stigma, fear, or misinformation surrounding COVID-19 that we haven’t discussed today?**
